# Tailor-made fermentation of sprouted wheat and barley flours and their application in bread making: A comprehensive comparison with conventional approaches in the baking industry

**DOI:** 10.1016/j.crfs.2025.101053

**Published:** 2025-04-12

**Authors:** Giuseppe Perri, Graziana Difonzo, Lorenzo Ciraldo, Federico Rametta, Gaia Gadaleta-Caldarola, Hana Ameur, Olga Nikoloudaki, Maria De Angelis, Francesco Caponio, Erica Pontonio

**Affiliations:** a Department of the Soil, Plant and Food Sciences (DiSSPA), University of Bari Aldo Moro, Via Amendola, 165/a, Bari, I-70126, Italy; b Faculty of Agricultural, Environmental and Food Sciences, Libera Universitá di Bolzano, Piazza Universitá, 5, 39100, Bolzano, Italy

**Keywords:** Lactic acid bacteria, Sprouted flours, Bioactive compounds, Baking industry, Tailor made food, Whole grains

## Abstract

This study investigates the development and application of type III sourdoughs, produced by fermenting sprouted wheat and barley flours with carefully selected lactic acid bacteria (LAB). Two optimized combinations of LAB strains were used: *Furfurilactobacillus rossiae* (CR5), *Weissella confusa* T6B10, and *Lactiplantibacillus plantarum* SB88.B4 for sprouted wheat flour; and *Leuconostoc pseudomesenteroides* DSM 20193, *L. plantarum* 7A, and F*. rossiae* (CR5) for sprouted barley flours. Fermentation resulted in substantial increases in peptide content (450 % in sprouted wheat flour-based sourdough and 520 % in sprouted barley flour-based sourdough) and phenolic compounds (344 % and 261 %, respectively), along with improved antioxidant activity (100 % in wheat and 40 % in barley). Among the experimental breads, those made with sprouted barley sourdough demonstrated the highest nutritional and functional benefits, including a highest content of dietary fiber, improved *in vitro* protein digestibility (IVPD, 81.14 %), a reduced predicted glycemic index (pGI, 84.78 %), and strong angiotensin-converting enzyme (ACE) inhibitory activity (73 %). The rheological behaviour of doughs incorporating novel type III sourdoughs was comparable to those containing type II wheat sourdoughs combined with enzymatyc enanchers, indicating their suitability for baking applications. Sensory evaluations highlighted that bread made with type III sourdough from sprouted wheat flour was appreciated for its enhanced crust and crumb colour, while bread made with sprouted barley sourdough stood out for its rich bran aroma, toasted notes, and balanced acidity. This study highlights the potential of targeted fermentation of sprouted flours as a key solution to address the growing demand for health-focused and eco-friendly innovations from both consumers and producers.

## Highlights

• Fermentation increases peptides, TFAA, and polyphenols in sprouted flours.
• Fermentation regulates flour enzymatic activity useful in bread making.
• Type III sourdoughs from sprouted flours enhance rheology.
• Fermentation boosts *In vitro* protein digestibility and ACE inhibitory activity.
• Sensory profile improved with type III sourdoughs.

## Graphical abstract

**Figure:**
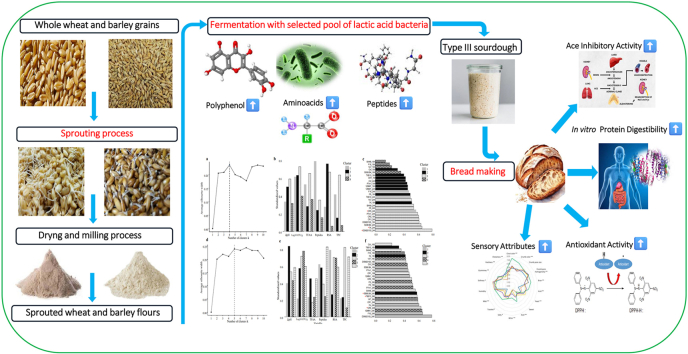

## 1 Introduction

Innovative technologies are required to shift towards a more sustainable and healthy diet, ensuring food safety and food security (Varzakas and Smaoui, 2024). In this context, functional foods have gained significant attention, with bakery products standing out as a promising category. These products offer a unique opportunity to carry essential nutrients, combining flavor, convenience, and health benefits (Guiné and Florença, 2024). Wheat flour, a main ingredient in most baked goods, is an important source of carbohydrates making up about 20 % of the world's daily energy intake and also provides substantial amounts of protein, vitamins (e.g., B vitamins), soluble dietary fiber, and phytochemicals (Wieser et al., 2020). However, refined wheat flour is missing some essential amino acids, such as lysine, methionine, and threonine, which lowers its protein quality compared to other grains (Hussain et al., 2020). Barley (*Hordeum vulgare* L.) is a good alternative because it has more soluble dietary fiber, especially β-glucans (5.12 % in barley flour versus 1.75 % in wheat), and higher levels of lysine, an essential amino acid important for human health (Abdullah et al., 2022). Adding cereals like barley to wheat flour-based baked goods increases fiber variety, supports gut health, and improves overall metabolic well-being. This also helps make food systems stronger and addresses the common lack of fiber in modern diets (Wang et al., 2022). Although barley shows nutritional promise, its bread-making potential is limited by technological challenges (Mariotti et al., 2014). Nevertheless, blends of barley and wheat flour have shown promising results in products like bread and cakes (Abdullah et al., 2022; Gujral et al., 2003). To enhance their functionality, advances in food science have further driven the use of sprouted grain flours, especially from wheat and barley, in bakery products (Abdi et al., 2024; Yaqoob et al., 2018; Marti et al., 2017). Sprouting is a sustainable, cost-effective process that enhances bioactive compounds and improves grain functionality, increasing nutrient digestibility and bioavailability (Perveen et al., 2024; Benincasa et al., 2019). One key advantage of sprouted flours is their potential to reduce the use of traditional enzymatic improvers, which are less cost-effective and sustainable, thereby increasing consumer acceptance and their applicability across various applications (Kehinde et al., 2022; Maqbool et al., 2024). Indeed, sprouting process activates hydrolytic enzymes like amylases and proteases (Benincasa et al., 2019), which, in low doses, can enhance leavening, crumb softness and shelf life of the products (Marti et al., 2017, 2018). However, if the sprouting process is not fully controlled, flours can bring, enzymatic activity and strong herbal flavors that limit their industrial applications (Peñaranda et al., 2021; Marti et al., 2018). Since the effects of germination on the characteristics of grains are not yet fully understood (Nonogaki et al., 2010; Nautiyal et al., 2023), the focus has now shifted to optimizing the use of sprouted flours to fully leverage their benefits without compromising dough performance or the sensory appeal of derived baked goods. In this regard, sourdough fermentation has emerged as a potential method to mitigate these challenges, enhancing the functional and sensory properties of sprouted flours in bakery applications (Gobbetti et al., 2019). Initial studies on fermenting sprouted flours with lactic acid bacteria (LAB) have shown promise in improving their qualities (Montemurro et al., 2019; Perri et al., 2023), yet no research has focused on selecting specific LAB strains tailored for these flours. This study aimed to fill this gap by testing 150 strains of LAB for their ability to ferment sprouted wheat and barley flour. The most effective twelve LAB strains were then combined into optimized pools to enhance fermentation. The goal was to develop new bio-ingredients (type III sourdough) from these sprouted flours, which were subsequently evaluated in bread-making trials. Breads were then assessed for their nutritional, technological, and sensory properties, with the performance of the novel bio-ingredients compared to conventional solutions used in the baking industry.

## 2 Materials and Methods

### 2.1 Grains and baking ingredients

Wheat (*Triticum aestivum* L.) and barley (*Hordeum vulgare* L.) grains were sourced from Molino Merano (Bolzano, Italy). The cSD (type II wheat fluor-based sourdough) and food grade calcium propionate (ADDCON, Germany) were supplied by Vallefiorita S.r.l. (Ostuni, Italy). The S500 CL enzyme enhancer was provided by Puratos S.r.l. (Brussels, Belgium), while extra virgin olive oil was obtained from Olearia Locantore (Andria, Italy).

### 2.2 Sprouting process of wheat and barley grains and chemical and biochemical analyses of flours

Wheat and barley grains were subjected to partial and assisted sprouting according to the protocol described by Montemurro et al. (2019). Both sprouted and non-sprouted grains were subsequently milled to obtain sprouted wheat flour (SWF), sprouted barley flour (SBF), whole wheat flour (WWF), and whole barley flour (WBF). For proximate composition analysis, protein content was measured following the UNI CEN ISO/TS 16634–2:2016 standard. Ash content was determined according to ISO 2171:2007, while dietary fiber was quantified using AOAC method 985.29 (Association of Official Analytical Chemists). Total lipids and total sugars were measured using the 62-C and 195-CH-29 methods from ISTISAN (1996/34, page 41, Met A). Available carbohydrates were calculated as 100 minus the sum of protein, lipids, ash, and fiber content. For biochemical characterization, water-soluble extracts (WSE) and methanol/water-soluble extracts (MWSE) were prepared following the procedures by Weiss et al. (1993) and Ameur et al. (2022), respectively. The WSE samples were analyzed for peptide concentration and total free amino acids (TFAA) using the O-phthalaldehyde and cadmium-ninhydrin assays, as per the methods of Church et al. (1983) and Doi et al. (1981). MWSE samples were used to determine total phenolic content (TPC) with the Folin-Ciocalteu method (Singleton and Rossi, 1965) and antioxidant activity, assessed via scavenging activity on the DPPH radical (Yu et al., 2003). The proximate composition and biochemical characteristics of both sprouted and non-sprouted flours are provided in Table S1.

### 2.3 Starter selection and fermentation setup

#### 2.3.1 Single-strain fermentation of sprouted wheat and barley flours

Starters were selected among 150 LAB strains, belonging to the Culture Collection of the Department of Soil, Plant and Food Sciences (University of Bari Aldo Moro, Italy), Faculty of Agricultural, Environmental and Food Sciences (Libera Universitá di Bolzano), and Leibniz Institute DSMZ (Germany). Functional (e.g., proteolytic activity, phytase and radical scavenging, in the methanolic extract) activities and pro-technological (kinetics of growth and acidification) features of LAB in their own isolation matrices (Nionelli et al., 2014, 2018a, 2018b; Pontonio et al., 2015; Rizzello et al., 2010a, 2016) were considered as criteria for a preliminary selection. Twelve selected LAB strains showing the best performing activity (e.g., higher cell density, proteolytic activities estimated as release of TFAA and peptides as well as the radical scavenging activity) were selected for further analyses to be performed *in situ* (sprouted wheat and barley flours). *Lactiplantibacillus plantarum* (strains CR1, 1A7, 12MM1, SD88.B4, 7A), *Pediococcus pentosaceus* I76, *Weissella confusa* (F16, BAN8, T6B10), *Furfurilactobacillus rossiae* (CR5), *Levilactobacillus brevis*(P7m1, and *Leuconostoc pseudomesenteroides* DSM 20193 were cultivated in De Man, Rogosa and Sharpe (MRS, Sigma-Aldrich, 69966, Dorset, UK) broth at 30 °C for 24 h, followed by cell harvesting via centrifugation at 10,000×*g* for 10 min at 4 °C, washing in 50 mM potassium phosphate buffer (pH 7.0), and resuspension in tap water. Each strain was inoculated into doughs prepared with sprouted wheat and barley flours (dough yield of 200, comprising 50 % w/w tap water and 50 % w/w flour) to reach a final cell density of ca. 7.0 Log cfu/g. Control samples included both non-inoculated dough and dough treated with chloramphenicol and cycloheximide (Sigma Aldrich, Germany) at a concentration of 0.1 g/L prior to incubation. Doughs were fermented at 30 °C for 16 and 24 h. LAB cell density, pH, and total titratable acidity (TTA) were measured prior (t0) and after 16 (t16) and 24 h (t24) of fermentation, following the methods reported by Rizzello et al. (2014). Additionally, at t0, t16, and t24, analyses were conducted to determine TFAA, peptides, TPC, and radical scavenging activity, as described in paragraph 2.2.

#### 2.3.2 Selection criteria for starter pools

Pro-technological properties such as pH variation (ΔpH) and LAB cell density growth (ΔLog cfu/g), and functional features like the content of TFAA, total peptides, TPC and the radical scavenging activity were used to select the best performing strains to be used as mixed starter for fermentation. Each variable was normalized (mean = 0, standard deviation = 1), and weighted composite scores were calculated with the following weights: ΔpH (0.15), LAB density (0.15), TFAA (0.20), peptides (0.20), DPPH (0.15), and TPC (0.15). K-means clustering and the Silhouette Method were used to optimize strain groupings, and the strain with the highest composite scores and distinct cluster groupings were selected for inclusion in 3 strain starter pools.

#### 2.3.3 Production and characterization of type III sourdoughs

Based on the selection, two ternary starter pools were used. *F. rossiae* (CR5), *W. confusa* (T6B10), and *L. plantarum* (SB88.B4) composing the first ternary starter pool, and *Leuc. pseudomesenteroides* (DSM, 20193), *L. plantarum* (7A), and *F. rossiae* (CR5) the second one were used for sprouted wheat flour-sprouted barley flour-based doughs, respectively. The selected strains were inoculated (ca. 7 Log cfu/g, 1:1:1) into the respective dough (DY 200) and the fermentation was carried out at 30 °C for 24 h. After fermentation, the sourdoughs were dried at 50 °C for approximately 24 h in a ventilated oven (Binder, Germany) until reaching a moisture content of 6.5 %. They were then milled with a laboratory mill (Braun AG, Type 4036, Frankfurt, Germany) to obtain a fine powder classified as type III sourdough and labeled as SW-sd and SB-sd for sprouted wheat and barley flours, respectively. Proximate composition and biochemical characteristics, including pH, TTA, total peptides, TFAA, TPC and radical scavenging activity were analyzed following the methods described in paragraphs 2.2 and 2.3.

### 2.4 Experimental breads making

A total of ten experimental bread formulations were developed, with their detailed compositions provided in Table 1. The dough yield was standardized at 190, maintaining a flour:water ratio of 52.5:47.5. The Control Wheat Bread (CWB) was made entirely from wheat flour, while the Sprouted Wheat Bread (SWB) replaced 1 % (w/w) of wheat flour with sprouted wheat flour (SWF). The Fermented Sprouted Wheat Bread (fSWB) incorporated 7.5 % (w/w) of type III SW-sd. The Sourdough Enzyme Wheat Bread (SEWB) contained 10 % (w/w) of type II wheat flour-based sourdough (cSD) and 0.5 % (w/w) of the S500 CL enzyme enhancer, whereas the Enzyme Wheat Bread (EWB) included only 0.5 % (w/w) of the S500 CL. For the formulations containing barley flour, the Control Barley Bread (CBB) replaced 7.5 % (w/w) of wheat flour with whole barley flour (WBF). The Sprouted Barley Bread (SBB) replaced 1 % (w/w) of wheat flour with sprouted barley flour (SBF), and the Fermented Sprouted Barley Bread (fSBB) incorporated 7.5 % (w/w) of type III SB-sd. The Sourdough Enzyme Barley Bread (SEBB) replaced 7 % (w/w) of wheat flour with whole barley flour, incorporated 10 % (w/w) of type II cSD (which accounted for 4.5 % of the total flour), and included 0.5 % (w/w) of the S500 CL. The Enzyme Barley Bread (EBB) substituted 7.5 % (w/w) of wheat flour with whole barley flour and also included 0.5 % (w/w) of the S500 CL. Each formulation was further supplemented with 0.5 % (w/w) olive oil, 0.1 % (w/w) calcium propionate, 1.2 % (w/w) salt, and 1.5 % (w/w) fresh baker's yeast as a leavening agent, which were not considered in the DY calculation. All ingredient percentages above expressed are based on total dough weight. The doughs were mixed using an Electrolux Assistant (EKM4000) mixer, divided into 500 g portions, shaped, and proofed for 120 min at 28 °C and 70 % relative humidity. After proofing, the loaves were baked at 180 °C for 1 h. Each formulation was tested in two independent trials, producing five loaves per batch.

**Table 1:** Formulation of the ten experimental breads. The formulations include variations of whole wheat and barley flours, incorporating sprouted flours, sourdoughs (SW-sd, SB-sd and cSD), enzyme additives (S500 CL), and other ingredients such as olive oil, salt, calcium propionate, and fresh baker's yeast. All formulations were prepared with a dough yield of 190.

	CWB		SWB		fSWB		SEWB		EWB		CBB		SBB		fSBB		SEBB		EBB	
Recipes	%d.b a	f.b.^b^	%d.b.	%f.b.	%d.b.	%f.b.	%d.b.	%f.b.	%d.b.	%f.b.	%d.b.	%f.b.	%d.b.	%f.b.	%d.b.	%f.b.	%d.b.	%f.b.	%d.b.	%f.b.
Whole wheat flour	52.5	100	52	99	45	85	47.5	90	52	99	45	85	52	99	45	85	40	77	44.5	84
Whole barley flour	–	–	–	–	–	–	–	–	–	–	7.5	15	–	–	–	–	7.5	14	7.5	15
Sprouted wheat flour	–	–	0.5	1	–	–	–	–	–	–	–	–	–	–	–	–	–	–	–	–
Sprouted barley flour	–	–	–	–	–	–	–	–	–	–	–	–	0.5	1	–	–	–	–	–	–
Water	47.5	90	47.5	90	47.5	90	42	79	47.5	90	47.5	90	47.5	90	47.5	90	42	79	47.5	90
SW-sd^c^	–	–	–	–	7.5	15	–	–	–	–	–	–	–	–	–	–	–	–	–	–
SB-sd^c^	–	–	–	–	–	–	–	–	–	–	–	–	–	–	7.5	15	–	–	–	–
cSD^d^	–	–	–	–	–	–	10	20	–	–	–	–	–	–	–	–	10	20	–	–

*Compostion of Type II cSD*																				
*Flour*	–	–	–	–	–	–	*4.5*	*9*	*-*	*-*	*-*	*-*	*-*	*-*	*-*	*-*	*4.5*	*9*	–	–
*Water*	–	–	–	–	–	–	*5.5*	*11*	*-*	*-*	*-*	*-*	*-*	*-*	*-*	*-*	*5.5*	*11*	–	–

S500 CL Enzyme enhancer	–	–	–	–	–	–	0.5	1	0.5	1	–	–	–	–	–	–	0.5	1	0.5	1
Salt	1.2	2.3	1.2	2.3	1.2	2.3	1.2	2.3	1.2	2.3	1.2	2.3	1.2	2.3	1.2	2.3	1.2	2.3	1.2	2.3
Olive oil	0.4	0.75	0.4	0.75	0.4	0.75	0.4	0.75	0.4	0.75	0.4	0.75	0.4	0.75	0.4	0.75	0.4	0.75	0.4	0.75
Calcium propionate	–	–	–	–	–	–	0.1	0.19	0.1	0.19	–	–	–	–	–	–	0.1	0.19	0.1	0.19
Fresh baker yeast	1.5	2.8	1.5	2.8	1.5	2.8	1.5	2.8	1.5	2.8	1.5	2.8	1.5	2.8	1.5	2.8	1.5	2.8	1.5	2.8
**Flour sum**^e^	**52.5**	**100**	**52.5**	**100**	**52.5**	**100**	**52.5**	**100**	**52.5**	**100**	**52.5**	**100**	**52.5**	**100**	**52.5**	**100**	**52.5**	**100**	**52.5**	**100**
**Water sum**^f^	**47.5**	**90**	**47.5**	**90**	**47.5**	**90**	**47.5**	**90**	**47.5**	**90**	**47.5**	**90**	**47.5**	**90**	**47.5**	**90**	**47.5**	**90**	**47.5**	**90**

a **d.b**., dough basis.

b **f.b**., flour basis.

c **SW-sd**, type III sourdough obtained from sprouted wheat flour**; SB-sd**, type III sourdough obtained from sprouted barley flour.

d **cSD** (*control sourdough*), type II sourdough (dough yield of 220, consisting of 55 % w/w water and 45 % w/w wheat flour).

e Flour sum of the recipe was calculated as the sum of flour from sourdough, flour used in baking and added enzyme enhancer S500 CL.

f Water sum was calculated as the sum of water from sourdough and water used in baking. For all breads the total amount of water was the same, 90 % of flour basis (f.b.).

### 2.5 Bread characterization

#### 2.5.1 Biochemical and nutritional analyses

The proximate composition of the breads was determined as described in paragraph 2.2, with the addition of moisture content determination using the ISO 712:2010 method. The water activity (a_w_) of the samples was measured using the Aqua Lab 4 TE water activity meter (Meter Group Inc., Pullman, WA, USA) following the manufacturer's instructions.

The pH, TTA, and TFAA were analyzed as described in paragraph 2.3. ACE inhibitory activity and β-glucan were analyzed using the ACE KIT-WST (Dojindo Molecular Technologies Inc. Japan) and K-BGLU kit (Megazyme, International Ireland Limited, Bray, Ireland) respectively, following the manufacturer's instructions. The *in vitro* protein digestibility (IVPD) was assessed using the method proposed by Akeson and Stahmann (1964). Breads underwent a sequential enzyme treatment, simulating *in vivo* digestion in the gastrointestinal tract. IVPD was expressed as the percentage of total protein solubilized following enzyme hydrolysis. Protein concentrations in both digested and non-digested fractions were determined using the Bradford method (Bradford, 1976). Starch hydrolysis index (HI) was determined as reported by De Angelis et al. (2007), using a D-glucose assay kit (GOPOD format, Megazyme) to detect the glucose release. The predicted glycemic index (pGI) was calculated using Capriles and Arêas' (2013) equation: pGI = 0.549 × HI + 39.71. TPC and antioxidant activity of the experimental breads were determined as described in paragraph 2.2.

#### 2.5.2 Physical and rheology analyses

The colorimetric determination was carried out on the bread's crust and crumb using a colorimeter CM-600d (Konica Minolta, Tokyo, Japan) and the software Spectramagic NX (Konica Minolta, Tokyo, Japan). As colour coordinates, lightness (L∗), redness (a∗, red-green), and yellowness (b∗, yellow-blue) were identified. All measurements were performed in triplicate.

The texture profile analysis (TPA) of each sample was carried out according to Pasqualone et al. (2019b), with some modifications. The analysis was performed on bread crumb (2 × 2 cm) using a texture analyzer Z1.0 TN (Zwick Roell, Ulm, Germany), equipped with a stainless-steel cylindrical probe (36 mm diameter) and a 50 N load cell. Two compressive cycles were performed at 1 mm/s probe compression rate and 50 % sample deformation in both the compression, with 5 s pause before second compression. At the end of compression, hardness, chewiness, springiness and cohesiveness were evaluated. Each sample was tested in three replicates. Data were acquired by the TestXPertII version 3.41 software (Zwick Roell, Ulm, Germany).

The rheological properties of the dough were evaluated using a HAAKE MARS iQ Air rheometer (Thermo Fisher Scientific, Waltham, Massachusetts, USA) equipped with parallel plate geometry (P35/Ti-02180932). Measurements were conducted at a constant temperature of 25 °C, with a 0.8 mm gap between the plates. The frequency-dependent behaviour of the samples was analyzed through an oscillatory frequency sweep, following the method described by Krystyjan et al. (2015), with minor modifications. The frequency ranged from 0.1 to 10 Hz, with a strain of 1 % to ensure the measurements remained within the linear viscoelastic region (De Angelis et al., 2024). The storage modulus (G′) and loss modulus (G″) were recorded as functions of frequency. Additionally, the modulus data were fitted to the Power Law model (Nilsson et al., 2023). For the dough the temperature sweep analysis was performed following the methodology described by Hesso et al. (2015), with some modifications. The dough was analyzed at a frequency of 1 Hz, with a 0.05 % strain (within the linear viscoelastic region) and a 2 mm gap between the plates. The temperature was increased from 25 °C to 98 °C at a rate of 3 °C/min, while the storage modulus (G′) and loss modulus (G″) (Pa) were recorded as functions of temperature. To prevent drying during the analysis, a sample holder was used to cover the dough (De Angelis et al., 2024). All measurements were performed in triplicate. The determination of specific volume was carried out by the procedure as described in the AACC method 10-05.01 (American Association of Cereal Chemists) (AACC, 2000).

#### 2.5.3 Sensory analysis

Sensory analysis of the experimental bread was conducted by a trained panel of twelve judges, aged **25 to 50 years**, at the University of Bari Aldo Maro (Italy). All judges were free from allergies or food intolerances and were regular consumers of bakery products. The sensory analysis followed the ethical guidelines of the laboratory as described from Pasqualone et al. (2019a) and informed consent was obtained from each panellist. A total of eighteen sensory descriptors were evaluated, reflecting the intensity of appearance, olfactory, taste, retro-olfactory and textural attributes, using a 10-point scale. Appearance attributes were evaluated indicating the crust colour intensity (0 = beige, 10 = dark brown), crumb colour (0 = beige, 10 = dark brown), crumb pore homogeneity (0 = inhomogeneous, 10 = homogeneous) and size of crumb pore (0 = 1–2 mm, 9 = 6–7 mm). The olfactometric descriptors were evaluated indicating the intensity of bran, acid and yeast odor notes; the intensity of sweet, acid, bitter and salty were evaluated by tasting while for retro-olfactory attributes the intensity of toasted and malt were evaluated. Finally, the intensity of texture attributes such as humidity, softness, gummines, hardness and chewiness were tested.

### 2.6 Statistical analysis

All data are expressed as means ± standard deviation (SD). Statistical significance between values was determined at p < 0.05 using analysis of variance (ANOVA), followed by Tukey's Honest Significant Difference (HSD) test, Fisher's Least Significant Difference (LSD) test, and Student's t-test for multiple comparisons. The statistical analyses were performed using Minitab Statistical Software (Minitab Inc., State College, PA, USA). Data processing and visualization for defining the starter pool, as described in paragraph 2.3.2, were carried out using R v4.3.1 statistical software. The graphics of the rheological data were created using GraphPad Prism version 9 (GraphPad Software, San Diego, CA, USA).

## 3 Results and discussion

### 3.1 Single-strain fermentations of sprouted wheat and barley flours

**Fig. 1:**
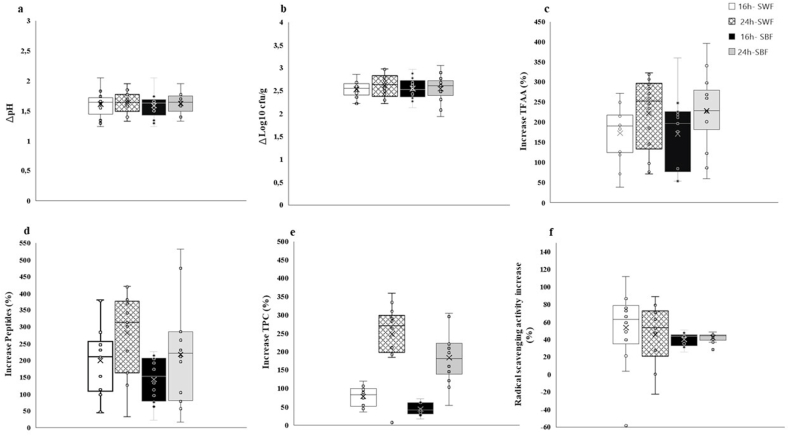
Boxplots illustrating the pro-technological (panels a–b) and functional (panels c–f) properties of twelve selected strains of lactic acid bacteria (LAB), including *Lactiplantibacillus plantarum* (CR1, 1A7, 12MM1, SD88.B4, 7A), *Pediococcus pentosaceus* I76, *Weissella confusa* (F16, BAN8, T6B10), *Furfurilactobacillus rossiae* CR5, *Levilactobacillus brevis* P7m1, and *Leuconostoc pseudomesenteroides* DSM 20193. Panels (a) and (b) illustrate acidification and growth performances of LAB strains. Panels (c–f) present the percentage increase in key functional parameters, including total free amino acid (TFAA) concentration (panel c), peptides (panel d), total phenolic compounds (TPC) (panel e), and radical scavenging activity (panel f). Each parameter was measured in dough (dough yield, DY 200) prepared with sprouted wheat flour (SWF) and sprouted barley flour (SBF), inoculated with each bacterial strain at a final cell density of 7 Log cfu/g, and fermented for 16 and 24 h at 30 °C. Results were compared to two controls: a non-inoculated sample and a sample treated with antibiotics, both incubated under same conditions. The boxplots display the median (line within the box), mean ( × ), and individual inner points (○). The box bounds represent the interquartile range (25th to 75th percentiles), while the whiskers extend to the 5th and 95th percentiles of the data.

Sprouting and fermentation are biological processes that significantly enhance the nutritional profile and physicochemical properties of grains (Wu and Xu, 2019). When applied to cereals, pseudocereals, and legume grains, these processes promote the release and bioavailability of bioactive compounds such as peptides, total free amino acids (TFAA), phenolic compounds, and soluble fibers, which offer numerous health benefits (Montemurro et al., 2019; Perri et al., 2023). According to Gänzle and Gobbetti (2023), LAB play a dominant role in sourdough fermentation, driving significant transformations in the final products. In this study, the twelve most performing LAB strains from prominent sourdough species were selected *in silico* and used to ferment doughs made from sprouted barley and wheat flours for 16 and 24 h at 30 °C. Key functional and technological indicators (as detailed in the Materials and Methods section 2.3.2) were monitored. To eliminate the influence of indigenous LAB and assess the intrinsic enzymatic activity of sprouted flours (Perri et al., 2020; Navarro et al., 2024), two control conditions were applied: not inoculated doughs and doughs treated with antibiotics (chloramphenicol and cycloheximide at 0.1 g/L) prior to incubation. Data on ΔpH and ΔLog cfu/g (Fig. 1, panels a and b) showed low variability across both matrices and fermentation times, except for a slight increase in the median ΔLog cfu/g values observed at 24 h compared to 16 h for both substrates. Several studies have indicated that prolonged fermentation enhances the proteolytic capacity of LAB, leading to a higher release of peptides and TFAA compared to shorter fermentation times (Gänzle et al., 2014; Reale et al., 2021; Rizzello et al., 2008; Gobbetti et al., 2008). Proteolysis is mediated by microbial enzymes, such as cell-envelope proteinases (CEP), which degrade proteins into oligopeptides. These oligopeptides are transported into the cytoplasm via the Oligopeptide Permease (Opp) system and further broken down into peptides and free amino acids by intracellular enzymes (Savijoki et al., 2006; Doeven et al., 2005). Bioactive peptides are primarily released during cell autolysis; however, certain species, such as *Leuconostoc* spp., possess a more extensive set of intracellular proteolytic enzymes, intensifying protein degradation, particularly in prolonged fermentations (Cibik and Chapot-Chartier, 2000; Pessione and Cirrincione, 2016). As shown in Fig. 1 (panels c and d), the distribution of data on the percentage increase in TFAA and peptides was similar between sprouted wheat flour (SWF) and sprouted barley flour (SBF), as were the differences associated with fermentation times. In both cases, the mean or median values obtained from 24-h fermentations were higher than the 75th percentile of the 16-h trials. Overall, SWF showed the highest increase of peptides at both 16 and 24 h of fermentation. Some outliers significantly extended the upper range of both key functional parameters. Specifically, *L. plantarum* (7A) and *Leuc. pseudomesenteroides* DSM 20193 after 24 h yielded the highest percentage increases in TFAA, at +323 % and +396 % in SWF and SBF, respectively. For peptides, the most effective strains were *W. confusa* (F16) in SWF and *F. rossiae* (CR5) for SBF, with increases of +421 % and +521 %, respectively. Phenolic compounds can be present in soluble form within the cytoplasm, or they are covalently bonded to the plant cell wall. Their increase in fermented products is linked to the action of bacterial enzymes, such as tannase, β-glucosidase, and feruloyl esterase, which release phenolics from the plant matrix (Filannino et al., 2015; Gustaw et al., 2021). Specifically, phenolic acid esters, such as chlorogenic acid, could be hydrolyzed by microbial cinnamoyl esterases, increasing the content of free phenolic acids, which have higher bioavailability (Filannino et al., 2018). These free phenolic acids can undergo further transformations by microbial enzymes: Phenolic Acid Decarboxylases (PAD), which decarboxylate phenolic acids, removing a carboxyl group (-COOH) and converting them into vinyl derivatives, and Phenolic Acid Reductases (PAR), which hydrogenate hydroxycinnamic acids, producing ethyl derivatives, both with enhanced bioactivity. These enzymatic mechanisms, primarily studied in LAB species from the *Lactiplantibacillus* and *Leuconostoc* genera (Fessard et al., 2017; Filannino et al., 2018), play a crucial role in understanding the observed variations in phenolic content and radical scavenging activity during fermentation. Here, all strains tested in both matrices demonstrated increases in TPC, with fermentation duration having a pronounced impact on phenolic compound release (Fig. 1, panel e). When fermentation was extended from 16 to 24 h, median increases shifted from 82 % to 42 % at 16 h to 181 % and 270 % for SWF and SBF, respectively. Specifically, *L. plantarum* (12MM1) and *F. rossiae* (CR5) resulted in increases of up to 360 % in wheat, while *L. plantarum* (7A) and *Leuc. pseudomesenteroides* (DSM, 20193) reached 300 % in barley. The radical scavenging activity exhibited considerable variability between the two substrates. Flours from sprouted barley displayed a narrower range of increases (25.71–50.73 %), with most strains showing modest improvements across both fermentation times (Fig. 1, panel f). In contrast, SWF doughs compared to t0 demonstrated a much broader range of percentage increases, from −58.14 % (DSM, 20193, 16h) to 112.05 %, with *Lev. brevis* P7m1 emerging as the most effective strain in both SWF and SBF based doughs after 16h of fermentation. Literature often emphasizes a direct correlation between the antioxidant activity of fermented plant-based products and their phenolic content (Sarıtaş et al., 2024; Adebo and Medina-Meza, 2020). However, *Lev. brevis* P7m1 did not show a significant average increase in phenolic content compared to other strains, suggesting that its superior antioxidant effects may be attributed to the release of other bioactive compounds (e.g., peptides), as previously hypothesized for this LAB species (Noureen et al., 2018). Control samples treated with antibiotics generally fell within or below the 25th percentile for most of the parameters analyzed, across both fermentation times. However, uninoculated doughs at 24 h showed significant increases of +213 %, +185 %, and +151 % for peptides, TFAA, and TPC, respectively, highlighting the ability of the indigenous microbiota in cereal flours to drive substantial substrate transformations (Gebre et al., 2024; Blandino et al., 2003).

### 3.2 Pool definition for SWF and SBF based doughs fermentation

**Fig. 2:**
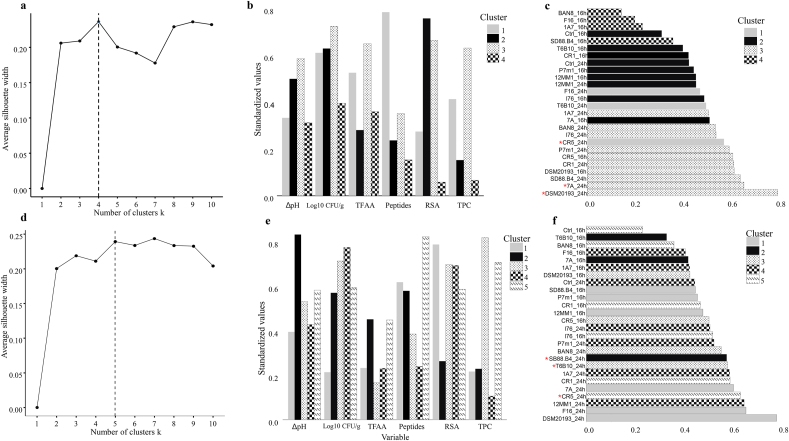
Optimal number of clusters, determined using K-means clustering based on normalized parameters and evaluated through the Silhouette method (panel a,d) Centroids of the clusters, extracted based on standardized values of ΔpH, LAB cell density (Log cfu/g), TFAA (*Total Free Amino acids*), Peptides, RSA (*Radical scavenging activity*), and TPC (*Total phenolic compounds*) (panel b; e); Composite scores of strains by cluster, obtained by combining composite score rankings with cluster groupings (panel c,f). Data refer to fermentations conducted on doughs (DY 200), prepared from sprouted barley flour (SBF) (panel a–c) and sprouted wheat flour (SWF) (panel d–f), and fermented for 16 and 24 h at 30 °C with the following strains: *Lactiplantibacillus plantarum* (CR1, 1A7, 12MM1, SD88.B4, 7A), *Pediococcus pentosaceus* (I76), *Weissella confusa* (F16, BAN8, T6B10), *Furfurilactobacillus rossiae* (CR5), *Levilactobacillus brevis* P7m1, and *Leuconostoc pseudomesenteroides* (DSM, 20193), inoculated at a final cell density of 7 Logcfu/g. Strains marked with a red asterisk (∗) in panel (c) and (f) were selected for multistrain fermentation of SBF and SWF based dough respectively. Control doughs, consisting of not inoculated dough incubated under the same conditions (Ctrl_16h and Ctrl_24h), were included in the data analysis.

Given the significant variability observed during single-strain fermentation, this study aimed to establish a starter pool for both substrates to enhance their quality across all evaluated parameters. Using methods like those reported in the literature (Fan et al., 2024), key fermentation parameters were standardized, normalized, and statistically analyzed. Each variable was normalized to ensure equal weighting in the analysis, and composite scores were calculated using predefined weightings. K-means clustering and the Silhouette Method were applied to categorize the strains, enabling a comprehensive performance evaluation and the identification of top-performing strains for each substrate (Fig. 2). For samples based on SBF, strains were grouped into four clusters (Fig. 2a). Cluster 3 included strains with high values for ΔpH, Log cfu/g, TFAA, and TPC, while Cluster 1 was notable for the highest peptide concentrations (Fig. 2b). Strain selection was thus based on composite scores and cluster analysis, incorporating two strains from Cluster 3, *Leuc. pseudomesenteroides* DSM 20193 and *L. plantarum* 7A, which exhibited strong performance across most properties, along with one strain from Cluster 1, *F. rossiae* CR5, to boost peptide concentrations (Fig. 2c). For samples based on SWF, strain selection showed greater diversity due to increased variability in the dataset; here, strains were grouped into five clusters (Fig. 2d). Cluster 2 strains exhibited the highest ΔpH values, while Cluster 3 strains demonstrated high Log cfu/g, radical scavenging activity, and TPC (Fig. 2e). Cluster 5 was distinguished by the highest peptide and TPC concentrations (Fig. 2e). A balanced selection strategy was employed, with one strain chosen from each of Clusters 2 (*L. plantarum*, SD88.B4), 3 (*W. confusa*, T6B10), and 5 (*F. rossiae*, CR5) to combine complementary properties (Fig. 2f). All strains selected for the pool definition are Generally Recognized as Safe (GRAS), apart from *W. confusa* (T6B10). Even though *Weissella* species are habitual components of microbial consortia in sourdough and have demonstrated significant technological and health promoting characteristics over time, they have not yet received GRAS status (De Vuyst et al., 2014; Fusco et al., 2023). Therefore, their use should be carefully considered in the context of future applications on industrial scale, as some strains may act as opportunistic pathogens in immuno-compromised individuals (Kamboj et al., 2015). In both cases, the optimal fermentation conditions were determined to be 24 h at 30 °C. Each selected pool was then used to initiate multi-starter fermentations on SWF- and SBF-based doughs, respectively, to produce type II sourdough.

### 3.3 Characterization of SWF and SBF based sourdoughs

An alternative approach in bread-making involves the use of type III sourdough, a dehydrated variant that serves as a functional bio-ingredient, enhancing both the nutritional and sensory qualities of baked goods. In contrast to traditional sourdough, type III sourdough offers superior stability and a longer shelf life, making it suitable for industrial applications (De Vuyst et al., 2023). In this study, type III sourdoughs were obtained from type II sourdoughs made from SWF and SBF, which were fermented for 24 h at 30 °C using the selected starter pools mentioned earlier. The resulting doughs were then dried at 50 °C for 24 h and ground into a fine powder, yielding two distinct type III sourdoughs: SW-sd from sprouted wheat and SB-sd from sprouted barley. Table 2 provides the chemical and biochemical-nutritional characterization of the two bio-ingredients. The chemical composition and quality of cereal flours vary due to the combined effects of cultivar-specific traits, weather conditions, and agricultural practices (Biel et al., 2020). Among cereals, barley is generally recognized as particularly high in fiber, especially soluble dietary fiber, which has enhanced its status as a valuable food ingredient over time (Biel et al., 2020). Here, the only significant difference in the chemical composition between the two sourdoughs was related to the fiber content, which was significantly higher (p < 0.05) in SB-sd than in SW-sd (Table 2). Regarding biochemical aspects, both sourdoughs achieved similar acidity levels by the end of fermentation, as no significant differences (p < 0.05) were observed in pH and total titratable acidity (Table 2). The fermentation quotient (FQ, the molar ratio of lactic acid to acetic acid) influences both the aroma profile of sourdough and the structure of baked products (De Luca et al., 2021). SW-sd and SB-sd showed approximately 250 mmol/kg of lactic acid and an acetic acid range from 10 to 17 mmol/kg, resulting in fermentation quotients of 14.17 for SW-sD and 22.72 for SB-sD, respectively. The recommended fermentation quotient is typically below 5.0, with a median value of 4.4 (Arora et al., 2021). Here, the QF of both sourdoughs were significantly higher (14.14 and 22.75 for SW-sd and SB-sd respectively), likely due to the drying process, which led to the volatilization of acetic acid, altering this balance (Pokajewicz et al., 2024). Ensuring sufficient accumulation of free amino acids (FAA) in sourdough is recommended to achieve high-quality products, as these compounds serve as essential precursors for flavor development (Arora et al., 2021). However, specifying an optimal range for amino acid content is challenging, as FAA concentration after sourdough fermentation can vary significantly due to factors such as flour type, fermentation conditions, and microbial strains used. Reported values ranged from 390 to 5000 mg/kg, with a median concentration of 1360 mg/kg (Arora et al., 2021). Notably, both SW-sd and SB-sd exhibited high levels of total FAA (TFAA), reaching 8217 mg/kg and 9772 mg/kg, respectively. The concentrations observed were approximately 12 times higher compared to their respective non-sprouted flours for wheat and around 7 times higher for barley, while they were nearly 3 times higher than in the sprouted flours for both wheat and barley (see Table 2 and Table S1). Sprouted grain flours are known to be inherently rich in bioactive compounds, as the sprouting process initiates proteolysis, releasing bioactive peptides, and triggers the synthesis of phenolic compounds as a defense response to the environment (Maqbool et al., 2024 –). However, the targeted fermentation approach developed in this study further enhances these properties significantly. Specifically, peptides content increased by 450 % and 520 % for SW-sd and SB-sd, respectively, when compared to their unfermented counterparts. Additionally, polyphenol content rose by 344 % for SW-sd and 261 % for SB-sd, along with a doubling of antioxidant activity for SW-sd and a 40 % increase for SB-sd (see Table 2 and Table S1). These substantial increases in bioactive compounds are consistent with previous findings, which suggest that fermentation not only complements but also synergistically amplifies the effects of sprouting, enhancing protein breakdown, phenolic synthesis and bioavailability (Shokoohi et al., 2015; Montemurro et al., 2019; Wu and Xu, 2019; Perri et al., 2023)

**Table 2:** Proximate composition, biochemical, nutritional, properties of type III sourdoughs produced from the fermentation of sprouted wheat flour (SW-sd) and sprouted barley flour (SB-sd). Doughs were prepared with a 1:1 flour-to-water ratio (dough yield 200) and fermented using selected ternary starter pools. For SW-sd, the selected strains included *Furfurilactobacillus rossiae* CR5, *Weissella confusa* T6B10, and *Lactiplantibacillus plantarum* SB88.B4. For SB-sd, the strains were *Leuconostoc pseudomesenteroides* DSM 20193, *L. plantarum* 7A, and *F. rossiae* CR5. Each dough was inoculated (7 Log cfu/g) and fermented for 24 h at 30 °C. The resulting sourdoughs were dried at 50 °C for 24 h. Data are expressed on dry weight.

	SW-sd	SB-sd
*Proximate composition*		
Carbohydrates (g/100g)	70.09 ± 2.12^a^	71.11 ± 1.89^a^
Protein (g/100g)	13.04 ± 0.84^a^	12.11 ± 0.52^a^
Fat (g/100g)	1.19 ± 0.03^a^	1.13 ± 0.05^a^
Total dietary fiber (g/100g)	9.60 ± 0.38^b^	11.58 ± 0.49^a^
Ash (g/100g)	1.31 ± 0.05^a^	1.45 ± 0.09^a^
*Biochemical and microbiological parameters*		
pH	3.93 ± 0.08^a^	3.96 ± 0.06^a^
TTA (mL NaOH)	47.12 ± 1.41^a^	50.91 ± 2.37^a^
Lactic acid (mmol/Kg)	239.70 ± 7.82^a^	246.85 ± 8.89^a^
Acetic acid (mmol/kg)	16.93 ± 0.67^a^	10.73 ± 0.41^b^
FQ	14.15 ± 0.43^b^	22.72 ± 0.77^a^
Total free aminoacids (TFAA) (mg/Kg)	8217 ± 285^b^	9772 ± 345^a^
Peptides (g/Kg)	28.70 ± 0.92^b^	39.38 ± 1.56^a^
Total phenolic compounds (mmol/Kg)	15.79 ± 0.72^b^	18.74 ± 0.89^a^
Radical scavenging activity (mmol BHT/Kg)	13.19 ± 0.49^a^	12.22 ± 0.61^a^

The data are the means of three independent experiments ± standard deviations (n = 3). ^a–b^ Values in the same row with different superscript letters differ significantly (p < 0.05).

### 3.4 Bread characterization

#### 3.4.1 Proximate composition and functional aspects

The quality of bread is influenced by various factors, including key ingredients, processing aids, additives, and specific baking methods (Collar et al., 2014). This study investigates the effects of experimental type III sourdoughs (SW-sd and SB-sd) compared with conventional bread-making ingredients, used under conditions consistent with previous research. Ten experimental formulations were developed: five with 100 % wheat flour and five incorporating a blend of wheat and barley flour in different proportions. In the CBB formulation, 15 % of the wheat flour was replaced with whole barley flour. This substitution level was chosen based on previous studies, which indicate that higher levels of barley inclusion can negatively affect dough performance and product acceptability (Dhingra et al., 2004; Ficco et al., 2023). Commercial enzyme blends, including xylanases, phytases, and α-amylases, are commonly used in the baking industry to improve dough properties and enhance the nutritional profile (Dahiya et al., 2020). For the EWB and EBB formulations, 1 % of the S500 CL enzyme enhancer was added, following the supplier's instructions. Research suggests that sprouted wheat and barley flours can be incorporated at levels ranging from 0.5 % to 2.5 % of the total flour weight without adversely affecting dough performance (Belcar et al., 2022; Marti et al., 2017). In this study, to explore the potential of sprouted flours as alternatives to commercial enzymes, 1 % of wheat flour was replaced with SWF or SBF in the SWB and SBB formulations. Type II sourdough, typically produced through single-stage fermentation as a liquid, is widely used in the baking industry (Siepmann et al., 2018). For the SEWB and SEBB formulations, a standard industrial procedure was followed, incorporating 20 % type II wheat sourdough and 1 % of the commercial enzyme blend (S500 CL). In accordance with validated inclusion levels in previous studies (Perri et al., 2023), type III sourdough (SW-sd and SB-sd) was added at 15 % in the fSWB and fSBB formulations. A notable effect of sprouting on the derived flours is the activation of endogenous enzymes, which leads to starch breakdown, an increase in sugar content, and a decrease in total dietary fiber (Ghavidel and Prakash, 2007; Benítez et al., 2013). Despite the low inclusion levels of alternative ingredients, significant differences in the chemical composition of the experimental breads were observed, particularly in sugar content and dietary fiber. The fSWB and fSBB formulations exhibited significantly higher sugar levels (1.93 and 1.78 g/100g) due to the high inclusion of sprouted flours through SW-sd and SB-sd, while fSWB showed lower fiber content compared to other formulations (Table 3). Conversely, formulations such as CBB, SEBB, and EBB, which contained higher proportions of whole barley flour, exhibited the highest fiber content (6.53, 6.24, and 6.44 g/100g, respectively). Lactic and acetic acids, the predominant metabolites in sourdough fermentation, typically result in higher total titratable acidity (TTA) and lower pH values in sourdough-enriched breads (Alkay et al., 2024). Bread containing type II and III sourdoughs (fSWB, fSBB, SEWB, and SEBB) showed significantly higher TTA and lower pH values (p < 0.05) compared to other formulations (Table 3). Barley stands out for its excellent protein profile among cereals, with high concentrations of essential amino acids, as reflected by the Digestible Indispensable Amino Acid Score (DIAAS) (Rani et al., 2021). Recent studies reveal a positive correlation between DIAAS and IVPD indices in plant-based protein sources (Sá et al., 2024; Krul et al., 2024). Barley-based formulations consistently demonstrated higher total free amino acid (TFAA) concentrations and IVPD values compared to their wheat-only counterparts. Specifically, the CBB formulation, when compared to its whole wheat-based counterpart (CWB), achieved both a higher TFAA content (950 mg/kg *vs*. 500 mg/kg) and a significantly higher (p < 0.05) IVPD index (72.95 % *vs*. 69.71 %). Significant chemical changes during sprouting include the reduction of condensed tannins, trypsin inhibitors, and phytic acid. These antinutritional compounds limit protein availability and digestibility, so their reduction enhances protein absorption (Singh et al., 2015). Germination also stimulates endogenous protease activity in flours, while LAB fermentation further promotes peptidase release, increasing the concentration of TFAA in the resulting flours (Singh et al., 2015; Gänzle, 2014). Formulations containing high levels of sprouted and fermented flours (fSBB and fSWB) exhibited the highest TFAA concentrations (1290 and 983.86 mg/kg), along with superior IVPD indices (81.14 % and 78.12 %), followed SEBB (968 mg/kg and 76.17 % for TFAA and IVPD respectively) (Table 3). Researchers are exploring strategies to address hypertension, a significant global health issue affecting approximately 1.3 billion adults worldwide (Baptiste, 2018). One approach involves incorporating angiotensin-converting enzyme (ACE) inhibitors into widely consumed foods, such as baked goods. Wholemeal cereal-based products are already recognized to contain bioactive compounds with antihypertensive properties (Garcia-Castro et al., 2022; Rizzello et al., 2008). Indeed, all the tested baked goods exhibited ACE inhibition activity greater than 60 % (Table 3). Prolamins in wheat and barley are rich in proline and hydrophobic amino acids, with ACE-inhibitory peptides often hidden within their primary sequence (Hu et al., 2011; Loponen et al., 2004). It has been demonstrated that the synergistic action of endogenous cereal, fungal, and microbial proteases is essential for releasing and activating these peptides (Zhao et al., 2013; Hu et al., 2011; Rizzello et al., 2008). This may help explain the significantly higher ACE inhibition activity (p < 0.05) observed in SEBB and fSBB (74.65 % and 73.30 % respectively), followed by fSWB and SEWB (72.43 % and 69.82 %). An interesting finding, considering the highly sensitivity of these peptides to the high temperatures used during baking (Buhlert et al., 2006). The wheat-only formulations (CWB, SWB, EWB) exhibited the highest starch hydrolysis index (HI) and predicted glycemic index (pGI) values (≥97 and ≥ 93, respectively) (Table 3). In contrast, the inclusion of both barley flour and sourdough led to significant reductions in these indices, with CBB and SEBB showing the lowest HI (87.51 % and 82.09 %) and pGI (87.75 and 84.78 %, respectively). This effect could be attributed to two factors: the biological acidification during sourdough fermentation and the higher β-glucan content in barley formulations, particularly in CBB (0.787 g/100g) and SEBB (0.676 g/100g). β-glucans create a protective barrier around starch granules, limiting amylase access and reducing starch hydrolysis, while lactic acid enhances the interaction between starch and gluten during baking, thereby reducing starch availability (Singla et al., 2024; Demirkesen-Bicak et al., 2021). The higher sugar levels combined with the reduction of β-glucan content in SWB (1.69 g/100g sugars, 0.074 g/100g β-glucans) and SBB (1.58 g/100g sugars, 0.234 g/100g β-glucans) likely account for their elevated HI (99.30 % and 99.97 %) and pGI (94.23 % and 94.59 %) values. This is probably due to the activity of α-amylase and β-glucanase, which are activated during germination and persisted throughout proofing (Capuano, 2017). These enzymes break down β-glucans and starch, facilitating sugar release and contributing to the increased glycemic response (Farzaneh et al., 2017; Rydzaka et al., 2024). Additionally, barley-based formulations exhibited higher total phenolic content (TPC) levels compared to wheat formulations. The CBB formulation showed a higher TPC content (0.464 mg GAE/g) compared to its wheat-based counterpart, CWB (0.219 mg GAE/g). The substitution of wheat flour with barley flour resulted in a significant increase in total phenolic content (p < 0.05) because barley naturally has a higher level of these compounds (Holtekjolen et al., 2008), due to the presence of the bran layer rich in phenolics and the increased bioavailability during the processing. Formulations fSWB and fSBB showed the highest TPC concentrations (0.537 and 0.833 mg GAE/g respectively), confirming what was already observed in paragraph 3.3 regarding the characterization of type III sourdoughs produced from the fermentation of sprouted wheat flour (SW-sd) and sprouted barley flour (SB-sd). Also, for the determination of antioxidant activity, the highest radical scavenging activity was observed for formulations fSWB and fSBB (0.876 and 0.736 mg GAE/g respectively) (Table 3). These results reinforce the findings discussed in paragraph 3.3, underscoring how the fermentation process enhances and complements the benefits achieved through the sprouting process (Perri et al., 2023). In Table 4, it can be observed that the water activity analysis did not show significant differences between the formulations (p < 0.05).

**Table 3:** Proximate composition, biochemical, and nutritional characteristics of experimental breads.

	CWB	SWB	fSWB	SEWB	EWB	CBB	SBB	fSBB	SEBB	EBB
*Proximate composition*										
Moisture (g/100g)	45.29 ± 2.42^a^	44.49 ± 1.62^a^	42.56 ± 1.89^a^	43.58 ± 1.84^a^	42.67 ± 1.99^a^	43.44 ± 1.54^a^	43.48 ± 1.13^a^	42.57 ± 1.87^a^	42.99 ± 2.22^a^	42.3 ± 1.90^a^
Carbohydrate (g/100g)	37.11 ± 1.97^a^	37.03 ± 1.73^a^	39.22 ± 1.84^a^	38.14 ± 1.84^a^	39.07 ± 1.79^a^	37.21 ± 1.92^a^	37.29 ± 2.29^a^	39.02 ± 1.34^a^	37.59 ± 2.67^a^	38.16 ± 1.42^a^
*Of wich sugars*	0.80 ± 0.04^d^	1.69 ± 0.04^b^	1.93 ± 0.09^a^	1.10 ± 0.09^c^	1.13 ± 0.06^c^	1.08 ± 0.06^c^	1.58 ± 0.07^b^	1.78 ± 0.08^a^	1.12 ± 0.09^c^	1.09 ± 0.07^c^
Protein (g/100g)	8.68 ± 0.276^a^	8.71 ± 0.326^a^	8.53 ± 0.217^a^	8.68 ± 0.299^a^	8.65 ± 0.455^a^	8.97 ± 0.207^a^	9.19 ± 0.354^a^	8.67 ± 0.144^a^	8.86 ± 0.282^a^	8.64 ± 0.213^a^
Total dietary fiber (g/100g)	5.64 ± 0.195^b^	5.69 ± 0.141^b^	5.12 ± 0.098^c^	5.66 ± 0.177^b^	5.44 ± 0.247^bc^	6.53 ± 0.285^a^	5.84 ± 0.162^b^	5.71 ± 0.146^b^	6.24 ± 0.195^a^	6.44 ± 0.183^a^
Fat (g/100g)	1.70 ± 0.05^a^	1.80 ± 0.06^a^	1.80 ± 0.07^a^	1.76 ± 0.09^a^	1.77 ± 0.11^a^	1.85 ± 0.09^a^	1.84 ± 0.07^a^	1.90 ± 0.14^a^	1.75 ± 0.09^a^	1.69 ± 0.09^a^
Ash (g/100g)	1.76 ± 0.04^a^	1.80 ± 0.06^a^	1.93 ± 0.14^a^	1.82 ± 0.09^a^	1.83 ± 0.07^a^	1.90 ± 0.08^a^	1.75 ± 0.08^a^	1.72 ± 0.06^a^	1.78 ± 0.04^a^	2.06 ± 0.13^a^
Energy (Kcal)	210 ± 7.61^a^	211 ± 8.09^a^	217 ± 8.34^a^	214 ± 5.57^a^	218 ± 6.06^a^	214 ± 7.12^a^	215 ± 7.44^a^	219 ± 8.89^a^	214 ± 7.77^a^	215 ± 6.67^a^

*Biochemical and Nutrtional Parameters*										

pH	5.91 ± 0.121^a^	5.97 ± 0.150^a^	5.11 ± 0.099^c^	5.15 ± 0.085^c^	5.78 ± 0.187^ab^	5.93 ± 0.111^a^	5.86 ± 0.143^ab^	5.06 ± 0.087^c^	5.25 ± 0.112^c^	5.63 ± 0.146^b^
TTA (mL NaOH)	3.00 ± 0.084^g^	3.07 ± 0.091^fg^	5.46 ± 0.135^b^	4.24 ± 0.102^d^	2.42 ± 0.065^h^	3.42 ± 0.093^e^	3.03 ± 0.088^fg^	6.02 ± 0.145^a^	5.03 ± 0.111^c^	3.22 ± 0.097^f^
TFAA (mg/Kg)	506.77 ± 16.23^g^	825.58 ± 23.12^cd^	983.86 ± 30.15^b^	645.56 ± 18.77^e^	570.65 ± 19.18^f^	951.24 ± 27.87^b^	848.54 ± 24.67^c^	1290.75 ± 34.00^a^	968.15 ± 25.91^b^	790.54 ± 22.13^d^
IVPD (%)	69.81 ± 0.88^e^	73.15 ± 1.01^d^	78.12 ± 1.27^b^	73.87 ± 1.13^cd^	71.09 ± 1.05^de^	72.95 ± 0.79^d^	75.09 ± 0.83^c^	81.14 ± 1.44^a^	76.17 ± 1.23^bc^	73.02 ± 0.96^d^
Ace inhibition (%)	66.56 ± 1.19^c^	66.31 ± 0.99^c^	72.43 ± 1.15^a^	69.82 ± 0.86^b^	63.08 ± 1.02^d^	63.30 ± 1.12^d^	62.58 ± 0.95^d^	73.30 ± 1.25^a^	74.65 ± 1.17^a^	67.31 ± 1.04^c^
HI (%)	97.10 ± 1.73^a^	99.30 ± 1.81^a^	87.65 ± 1.17^c^	93.15 ± 1.25^b^	97.51 ± 1.93^a^	87.51 ± 1.09^c^	99.97 ± 1.66^a^	88.65 ± 1.11^c^	82.09 ± 0.95^d^	93.25 ± 1.23^b^
pGI	93.02 ± 1.03^ab^	94.23 ± 1.24^a^	87.83 ± 1.27^c^	90.85 ± 1.35^b^	93.24 ± 1.39^a^	87.75 ± 1.05^c^	94.59 ± 1.38^a^	88.38 ± 1.07^c^	84.78 ± 0.96^d^	90.90 ± 1.13^b^
β-glucan (g/100g)	0.227 ± 0.003^e^	0.074 ± 0.001^i^	0.007 ± 0.001^j^	0.167 ± 0.002^g^	0.208 ± 0.002^f^	0.787 ± 0.010^b^	0.234 ± 0.002^d^	0.082 ± 0.001^h^	0.676 ± 0.011^a^	0.609 ± 0.009^c^
TPC (mg GAE/g)	0.219 ± 0.007^e^	0.233 ± 0.012^e^	0.537 ± 0.003^c^	0.263 ± 0.006^e^	0.219 ± 0.007^e^	0.464 ± 0.015^d^	0.482 ± 0.004^d^	0.833 ± 0.012^a^	0.698 ± 0.022^b^	0.624 ± 0.022^b^
DPPH (μmoli TE/g)	0.192 ± 0.01^e^	0.288 ± 0.006^d^	0.876 ± 0.018^a^	0.263 ± 0.014^d^	0.257 ± 0.014^d^	0.162 ± 0.015^e^	0.307 ± 0.017^c^	0.744 ± 0.019^b^	0.650 ± 0.006^b^	0.653 ± 0.01^b^

TFAA (*Total Free Aminoacids*); IVPD (*In Vitro Protein Digestibility*); HI (*Starch Hydrolysis Index*); pGI (*Predicted Glycemic Index*); total phenol content (TPC); DPPH assays of bread. The data are the means of three independent experiments ± standard deviations (n = 3). ^a–h^ Values in the same row with different superscript letters differ significantly (p < 0.05).

**Tables 4:** Colorimetric indices, activity water (A_w_) and specific volume (g/mL) of experimental breads.

	CWB	SWB	fSWB	SEWB	EWB	CBB	SBB	fSBB	SEBB	EBB
**Crust**										
L∗	61.45 ± 0.65a	47.31 ± 0.83c	45.82 ± 0.83c	45.63 ± 0.79c	54.06 ± 0.93b	45.36 ± 0.42a	42.03 ± 0.72b	40.77 ± 0.93c	44.23 ± 1.52a	43.46 ± 1.45^bc^
a∗	7.57 ± 0.84c	10.6 ± 0.9 ab	11.25 ± 0.66a	9.91 ± 0.58 ab	8.85 ± 0.75b	7.84 ± 0.22b	10.18 ± 0.93 ab	11.55 ± 0.48a	9.18 ± 0.92 ab	9.51 ± 0.87^b^
b∗	24.29 ± 0.6 ab	25.01 ± 0.66a	25.97 ± 0.72a	22.78 ± 0.52 ab	23.14 ± 0.72 ab	21.82 ± 0.52a	18.64 ± 0.39a	24.94 ± 0.38 ab	22.39 ± 1.15a	25.87 ± 1.02^ab^
Crumb										
L∗	55.06 ± 0.9a	45.53 ± 0.64b	42.95 ± 0.83b	45.66 ± 0.98b	45.53 ± 0.98b	45.07 ± 0.66 ab	48.25 ± 0.17a	43.35 ± 1.3 ab	44.39 ± 0.29 ab	48.25 ± 0.56^a^
a∗	5.43 ± 0.26b	5.52 ± 0.36b	6.03 ± 0.66a	5.33 ± 0.58b	5.16 ± 0.37b	4.49 ± 0.54b	5.2 ± 0.39b	7.32 ± 0.28a	4.82 ± 0.5b	5.2 ± 0.67^b^
b∗	20.22 ± 0.32a	19.08 ± 0.92a	19.78 ± 0.72a	19.51 ± 0.87a	19.61 ± 0.94a	17.2 ± 0.49a	19.06 ± 0.86a	21.81 ± 0.75a	18.46 ± 0.87a	19.06 ± 0.52^a^

Aw	0.98±0a	0.96±0a	0.98±0a	0.98±0a	0.98±0a	0.98±0a	0.98±0a	0.97±0a	0.96±0a	0.98±0^a^
Specific volume (mL/g)	2.19 ± 0.02a	2.18 ± 0.01a	2.04 ± 0.04a	2.18 ± 0.04a	2.27 ± 0.09a	2.1 ± 0.12a	1.69 ± 0.2a	2.18 ± 0.03a	1.99 ± 0.13a	2.19 ± 0.14^a^

The data are the means of three independent experiments ± standard deviations (n = 3). ^a–h^ Values in the same row with different superscript letters differ significantly (p < 0.05).

#### 3.4.2 Rheological properties of the doughs

**Fig. 3:**
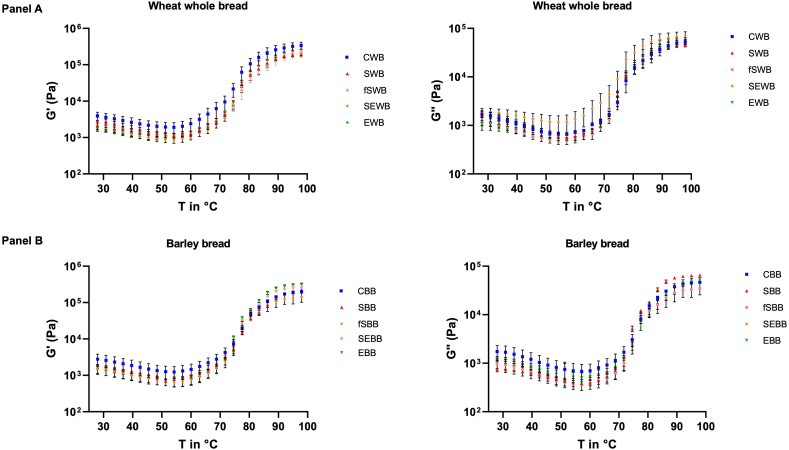
Trend of storage (G′) and loss (G″) modulus as a function of the temperature (°C) of the experimental doughs, (Panel A) Control Wheat Bread (CWB), Sprouted Wheat Bread (SWB), Fermented Sprouted Wheat Bread (fSWB), Sourdough Enzyme Wheat Bread (SEWB), and Enzyme Wheat Bread (EWB). (Panel B) Control Barley Bread (CBB), Sprouted Barley Bread (SBB), Fermented Sprouted Barley Bread (fSBB), Sourdough Enzyme Barley Bread (SEBB), and Enzyme Barley Bread (EBB).

**Fig. 4:**
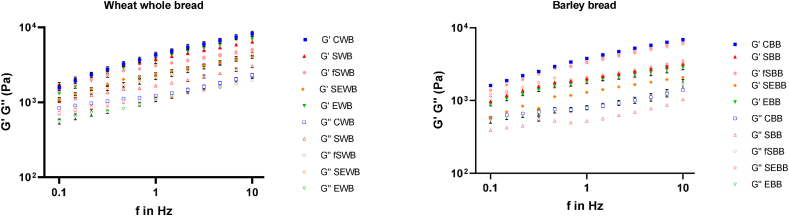
Trend of storage (G′) and loss (G″) modulus as a function of the linear frequency (Hz) of the experimental doughs. Filled symbols indicate measurement of storage (G′) modulus, while empty symbols indicate measurement of loss (G″) modulus, Control Wheat Bread (CWB), Sprouted Wheat Bread (SWB), Fermented Sprouted Wheat Bread (fSWB), Sourdough Enzyme Wheat Bread (SEWB), and Enzyme Wheat Bread (EWB), Control Barley Bread (CBB), Sprouted Barley Bread (SBB), Fermented Sprouted Barley Bread (fSBB), Sourdough Enzyme Barley Bread (SEBB), and Enzyme Barley Bread (EBB).

To better understand the rheological differences of the breads, the rheological properties of the doughs were estimated (Fig. 3 and Table 5). The viscoelastic properties of the doughs were examined through rheological analysis, which included temperature sweep tests and oscillatory frequency analysis. A frequency sweep analysis was conducted to assess the viscoelastic properties of all the dough samples across non-destructive deformation ranges, simulating short-time scales (high frequency) and long-time scales (low frequency) (Fig. 4) (Ramli et al., 2022). For both wheat-based and barley-based doughs, G′ was consistently higher than G″, indicating that the dough samples exhibited viscoelastic behaviour with dominant solid-like characteristics (Amemiya and Menjivar, 1992). When G′ > G″ (tan δ < 1), the material behaves predominantly as a gel or solid-like substance, whereas G′ < G″ (tan δ > 1) suggests a more liquid-like behaviour. As observed, G′ remained higher than G″ across all frequency ranges, highlighting the dominance of elastic properties over viscous properties in the dough samples (Upadhyay et al., 2012). Both moduli exhibited a slight dependence on frequency, showing a moderate increase as the frequency increased (Fig. 3). In Table 5 it can be observed that the lowest G′ values are represented by the SEWB sample for wheat-doughs and the SEBB sample for barley-based doughs, both of which include the use of sourdough in their formulation. Clarke et al. (2002) and Angioloni et al. (2006), who investigated the impact of sourdough fermentation on the fundamental rheological properties of wheat dough, reported that the incorporation of sourdough resulted in a less elastic bread dough. Hammes and Gänzle (1998) and Thiele et al. (2002) highlighted that the sourdough fermentation process significantly impacts dough properties, further supporting our observed trend. It was important to evaluate the rheological behaviour of the doughs to understand and optimize the physical and mechanical properties that influence the bread-making process and the quality of the final product.

**Table 5:** Rheological properties of dough at the frequency of equal to 1 Hz.

	CWB	SWB	fSWB	SEWB	EWB	CBB	SBB	fSBB	SEBB	EBB
G’	4298.5 ± 50a	3732.5 ± 44.5 ab	3079 ± 111 ab	2370.5 ± 110.5b	3598 ± 74 ab	3795 ± 73 ab	1999 ± 40bc	3365.5 ± 67.5a	1294±7c	1844.5 ± 77.5^c^
G’’	1233.5 ± 45.5a	1669 ± 55a	1134.5 ± 53.5a	2180 ± 79a	1095.5 ± 76.5a	788.75 ± 28.25b	524.2 ± 4.1b	2077 ± 39a	1865.5 ± 175.5b	798.8 ± 53.7^a^
tan δ	0.40 ± 0.00c	0.45 ± 0.01b	0.48 ± 0.02b	0.52 ± 0.01a	0.48 ± 0.02b	0.39 ± 0.01b	0.41 ± 0.01b	0.54 ± 0.01a	0.54 ± 0.03a	0.44 ± 0.00^b^

The data are the means of three independent experiments ± standard deviations (n = 3). ^a–h^ Values in the same row with different superscript letters differ significantly (p < 0.05).

Fig. 3 depicts the temperature-dependent behaviour of the dough, showing the G′ and G″ moduli as functions of temperature. A temperature sweep analysis was performed to replicate the baking process and examine the key rheological phenomena and structural transformations in the dough, offering valuable insights into the viscoelastic changes occurring during cooking (De Angelis et al., 2024). For this analysis as well, G′ was always higher than G″ for both wheat and barley-based doughs, indicating the predominance of elastic-like behaviour from the early stage of measurement. During the early stages of heating, the starch gradually gelatinized as the starch granules began to rupture and release absorbed water, resulting in a slight decrease in G′ and G″ (Hao et al., 2008; Marchini et al., 2021). As the gelatinization temperature of the starch was reached, gelatinization speeded up considerably, resulting in the observed rapid increases in G′ and G″. The action of α-amylase intensifies starch degradation, enhancing the gelatinization capacity of the flour mixture (Atudorei et al., 2021). The behaviour of the samples, both for barley-based and wheat-based doughs had very similar variations with temperature.

#### 3.4.3 Colour and textural evaluations

The colour parameters of the experimental breads are shown in Table 4. It's evident that barley-based breads have lower crust brightness (L∗) values across all samples compared to wheat-based breads. This could be attributed to the reduced gluten content in the barley formulations. Since the resulting system is unable to retain moisture effectively, there is an increase moisture loss from the crust. Consequently, the surface of the sample is not smooth and homogeneous, which may also reduce the crust's brightness (Komeili et al., 2014). Regarding the internal differences among the various formulations considering the crust and the crumb, it can be observed that the redness (a∗) shows significantly higher values in formulations using germinated flour and type III sourdoughs, and a decrease in brightness (L∗), both for wheat-based and barley-based breads. Similar results were found by Baranzelli et al. (2018), which highlight that the use of sprouted flours in bread production led to decreased brightness (L∗) and increased redness (a∗), while yellowness (b∗) remained unchanged. Johnston et al. (2019) also reported that the crumb of sprouted grain bread was less bright than that of non-sprouted loaves. Conversely, the analysis of specific volume did not show significant differences (p < 0.05) between the formulations, for the wheat-based and barley-based bread.

The study of texture parameters, such as hardness, chewiness, springiness, and cohesiveness of the experimental breads, is shown in Table 6. The results show that the formulations with partial substitution using sprouted flour (SWB and SBB) show lower values of cohesiveness, hardness, springiness and chewiness compared to the other formulations. The observed changes in texture properties may be attributed to the reduction in gluten content resulting from the addition of sprouted flour (Sun et al., 2023). Indeed, a study reports that the decrease in the relative crystallinity of starch could be due to the activation of some enzymes during germination, resulting in a disruption of the double-helix structures and the degradation of starch amylose and amylopectin this results in a less elastic and cohesive structure, lower hardness, and a bread that requires less force to be chewed. (Li et al., 2017; Waleed et al., 2021). Additionally, the results also demonstrate that both formulations (SEWB and SEBB) incorporating cSD (type II wheat flour-based sourdough) exhibit the highest hardness values (8.12 ± 0.14 and 8.24 ± 0.01 N, respectively). A study reported that hardness values increase as more sourdough is added (Siepmann et al., 2019). In fact, sourdough, thanks to its unique biochemical processes, creates a more compact and rigid matrix, which results in higher hardness values (Siepmann et al., 2019; Arendt et al., 2007).

**Table 6:** Textural parameters of experimental bread.

	CWB	SWB	fSWB	SEWB	EWB	CBB	SBB	fSBB	SEBB	EBB
Hardness (N)	6.78 ± 0.07^b^	5.6 ± 0.05^bc^	5.51 ± 0.07^c^	8.12 ± 0.14^a^	6.64 ± 0.06^b^	6.98 ± 0.08^b^	6.09 ± 0.05^b^	6.69 ± 0.07^b^	8.24 ± 0.01^a^	6.13 ± 0.03^b^
Chewiness (N)	4.7 ± 0.15^a^	2.06 ± 0.02^bc^	2.61 ± 0.03^b^	2.84 ± 0.08^b^	2.09 ± 0.06^c^	2.6 ± 0.07^b^	1.46 ± 0.05^c^	2.31 ± 0.08^b^	3.71 ± 0.07^a^	2.84 ± 0.02^b^
Springiness	0.88 ± 0.01^a^	0.71±0^c^	0.84 ± 0.01^ab^	0.86 ± 0.02^ab^	0.78±0^bc^	0.81±0^ab^	0.65 ± 0.03^c^	0.76 ± 0.02^b^	0.87 ± 0.02^a^	0.83 ± 0.01^a^
Cohesiveness	0.47 ± 0.04^b^	0.42 ± 0.02^b^	0.55 ± 0.01^a^	0.44 ± 0.02^b^	0.51 ± 0.02^ab^	0.53 ± 0.01^a^	0.36 ± 0.01^b^	0.48 ± 0.02^ab^	0.51 ± 0.02^a^	0.48 ± 0.01^ab^

The data are the means of three independent experiments ± standard deviations (n = 3). ^a–h^ Values in the same row with different superscript letters differ significantly (p < 0.05).

#### 3.4.4 Sensory properties

**Fig. 5:**
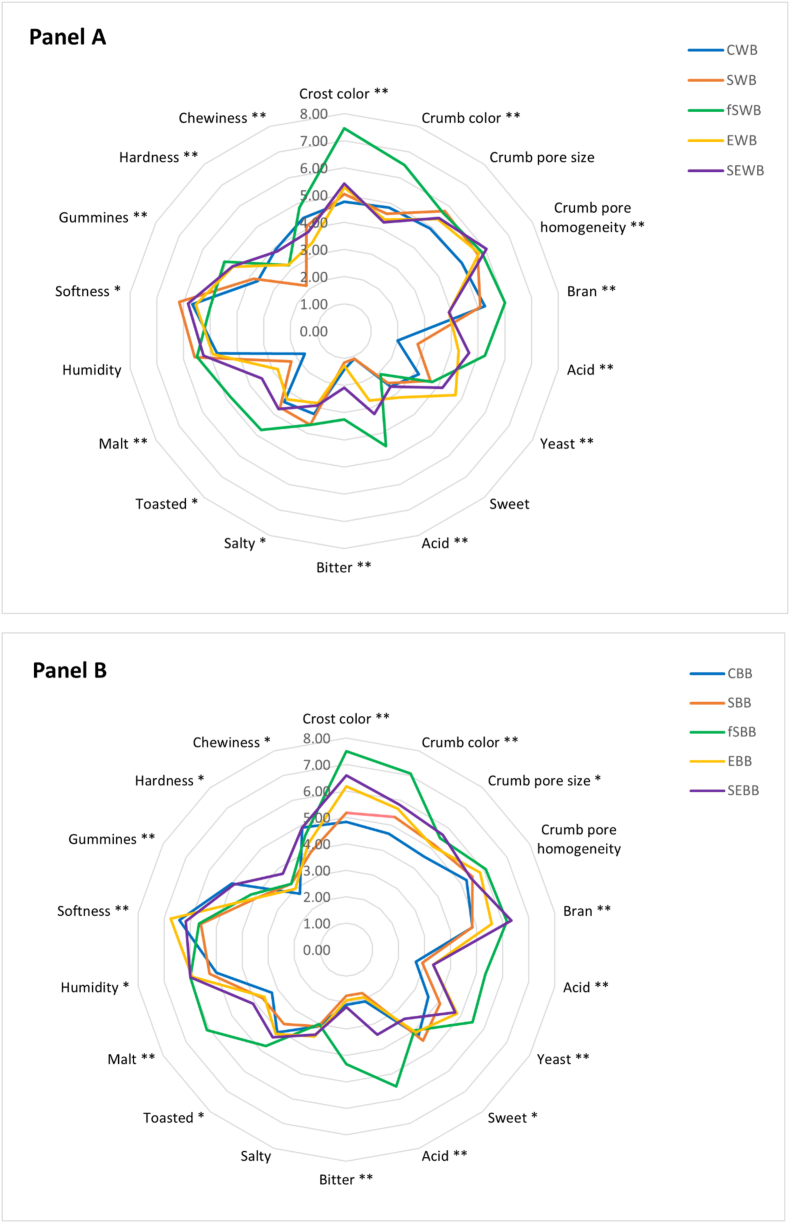
Spider-chart of the sensory analysis of the experimental breads. The data are the means of twelve independent experiments ± standard deviations (n = 12). a–h ∗∗ mean significant difference with p < 0.001 of experimental breads; ∗ mean significant difference for the experimental bread with p < 0.05. The colour-coded lines represent the different experimental breads: (Panel A) Control Wheat Bread (CWB, blue), Sprouted Wheat Bread (SWB, orange), Fermented Sprouted Wheat Bread (fSWB, green), Sourdough Enzyme Wheat Bread (SEWB, yellow), and Enzyme Wheat Bread (EWB, purple); (Panel B) Control Barley Bread (CBB, blue), Sprouted Barley Bread (SBB, orange), Fermented Sprouted Barley Bread (fSBB, green), Sourdough Enzyme Barley Bread (SEBB, yellow), and Enzyme Barley Bread (EBB, purple).

The sensory characteristics of wheat- and barley-based breads were evaluated (Fig. 5, Panel A and B). The analysis focused on various sensory attributes, including: (i) appearance, with assessments of crust colour intensity, crumb colour, homogeneity of crumb pores, and pore size; (ii) aroma, covering the intensity of bran, acidity, and yeast notes; (iii) taste-olfactory elements, such as the strength of toasted and malted flavours; (iv) taste, which examined the intensity of sweet, sour, bitter, and salty flavours; and (v) textural properties, including moisture content, softness, gumminess, hardness, and chewiness. The wheat-based bread with the fSWB formulation showed significant differences from other formulations, with a p-value of 0.001 (Fig. 5, Panel A). These differences were observed in sensory attributes such as crust colour, crumb colour, bran aroma, acidity, and bitterness. The distinct sensory profile of this bread can be attributed to the specific ingredient used, which influenced both its visual and flavour characteristics. Similarly, in barley-based breads, the fSBB formulation exhibited notable differences in attributes like crust colour, crumb colour, bran aroma, acidic and yeast notes, sour taste, and toasted retro-olfactory characteristics (Fig. 5, Panel B). These findings can be traced back to the use of type III sourdoughs made from fermented sprouted wheat (SW-sd) and sprouted barley (SB-sd) flours in the bread formulations. The results are consistent with those of instrumental analyses.

## 4 Conclusion

This study successfully developed an innovative fermentation protocol to enhance the value of sprouted wheat and barley flours, ingredients of growing interest in the baking industry. Guided fermentation with tailored selected ternary pools of LAB led to fermented flours with significantly improved nutritional profiles, marked by substantial increases in peptides, total free amino acids (TFAA), polyphenols, and antioxidant activity. Dehydrating these fermented flours yielded type III sourdoughs which, when used at 7.5 % of dough weight to produce experimental breads, delivered notable nutritional benefits, including enhanced *In vitro* protein digestibility (IVPD), ACE inhibitory activity, and a lower predicted Glycemic Index, with particularly pronounced effects in barley-based formulations. This approach addressed key challenges in utilizing sprouted flour by a controlled exploitation of their intrinsic enzymatic activity. It unlocked their full nutritional potential and facilitated the creation of a single ingredient capable of achieving both nutritional and technological outcomes that typically require the combined use of multiple enhancers in the baking industry. These findings mark a significant step toward sustainable solutions for the baking industry. Future research should explore the application of these biotechnological processes to alternative raw materials, such as pseudo-cereals and legumes. However, several challenges remain before they can be widely adopted on an industrial scale. A deeper understanding of the persistence of bioactivity post-digestion is essential, as well as consumer acceptance studies, which are key to evaluating the practicality of these innovations across different markets and social contexts. Moreover, while fermentation benefits from the widespread availability of pilot and industrial-scale equipment, scaling up germination is more challenging. Overcoming these barriers will be crucial for ensuring long-term viability and commercial scalability.

## CRediT authorship contribution statement

**Giuseppe Perri:** Writing – original draft, Investigation, Validation, Supervision, Methodology, Formal analysis, Data curation. **Graziana Difonzo:** Writing – original draft, Investigation, Validation, Supervision, Methodology, Formal analysis, Data curation. **Lorenzo Ciraldo:** Investigation, Validation, Formal analysis, Data curation. **Federico Rametta:** Investigation, Validation, Formal analysis, Data curation. **Gaia Gadaleta-Caldarola:** Validation, Formal analysis, Data curation. **Hana Ameur:** Validation, Formal analysis, Data curation. **Olga Nikoloudaki:** Validation, Formal analysis, Data curation. **Maria De Angelis:** Writing – review & editing, Funding acquisition. **Francesco Caponio:** Writing – review & editing, Funding acquisition. **Erica Pontonio:** Writing – review & editing, Writing – original draft, Supervision, Resources, Project administration, Funding acquisition, Conceptualization.

## Funding

This work was funded by PON Research and Innovation 2014–2020 and FSC - INTEGRI Project, ARS01_00188. CUP: B94I20000470005.

## Declaration of competing interest

The authors declare that they have no known competing financial interests or personal relationships that could have appeared to influence the work reported in this paper.

## Data Availability

No data was used for the research described in the article.
