# Supplementary material for: Tailor-made fermentation of sprouted wheat and barley flours and their application in bread making: A comprehensive comparison with conventional approaches in the baking industry
Source: Curr Res Food Sci. 2025 Apr 12;10:101053. doi: 10.1016/j.crfs.2025.101053 (PMC12022488; doi:10.1016/j.crfs.2025.101053)
Supplement: Multimedia component 1 [file mmc1.doc]

**Table S1 Proximate composition and biochemical parameters of whole and sprouted wheat and barley flours. Data are expressed on dry wieght basis.**

|  | **Whole wheat flour** | **Sprouted wheat flour** | **Whole barley flour** | **Sprouted barley flour** |
| --- | --- | --- | --- | --- |
| Carbohydrate (g/100g) | 67.59 ± 1.11^a^ | 74.08 ± 0.94^c^ | 62.49 ± 1.14^b^ | 71.14 ± 1.05^d^ |
| *of which sugar* | 5.54 ± 0.87^d^ | 31.58 ± 1.05^b^ | 7.95 ± 1.02^c^ | 38.61 ± 1.18^a^ |
| Protein (g/100g) | 15.46 ± 0.95^a^ | 15.34 0.29^a^ | 14.02 ± 0.66^a^ | 14.61 ± 0.82^a^ |
| Total dietary fiber (g/100g) | 14.03 ± 0.36^b^ | 10.58 ± 0.21^d^ | 19.52 ± 0.32^a^ | 12.64 ± 0.18^c^ |
| Fat (g/100g) | 1.11 ± 0.06^b^ | 0.87 ± 0.03^c^ | 0.88 ± 0.04^c^ | 1.50 ± 0.05^a^ |
| Ash (g/100g) | 1.45 ± 0.10^b^ | 1.31 ± 0.09^b^ | 2.46 ± 0.16^a^ | 2.12 ± 0.10^a^ |
| *Biochemical parameteres* | | | | |
| TFAA (mg/Kg) | 679 ± 16.97^d^ | 2850 ± 79.08^b^ | 1391 ± 27.27^c^ | 3228 ± 91.23^a^ |
| Peptides (g/Kg) | 2.46 ± 0.05^d^ | 5.28 ± 0.14^b^ | 3.87 ± 0.11^c^ | 6.26 ± 0.19^a^ |
| TPC (mmol/Kg) | 1.37 ± 0.03^d^ | 3.55 ± 0.09^b^ | 2.33 ± 0.06^c^ | 5.19 ± 0.16^a^ |
| Radical scavenging acitivty (mmol BHT/Kg) | 2.17 ± 0.04^d^ | 6.75 ± 0.19^c^ | 6.92 ± 0.19^b^ | 8.73 ± 0.29^a^ |

The data are the means of three independent experiments ± standard deviations (n = 3). ^a–d^ Values in the same row with different superscript letters differ significantly (p < 0.05). TFAA (*Total Free Aminoacids*), TPC (*Total Phenolic Compounds*).
